# Wernicke encephalopathy in a patient with liver failure: Clinical case report

**DOI:** 10.1097/MD.0000000000003651

**Published:** 2016-07-08

**Authors:** Pan Zhao, Yanling Zhao, Zhenman Wei, Jing Chen, Lilong Yan

**Affiliations:** a Clinical Trial Center, Liver Failure Therapy and Research Center; b Division of Pharmacology, Institute of Infectious Diseases, Beijing 302 Hospital (PLA 302 Hospital), Beijing, China.

**Keywords:** differential diagnosis, liver failure, Wernicke encephalopathy

## Abstract

Early recognition and diagnosis of Wernicke encephalopathy is pivotal for the prognosis of this medical emergency, especially in patients with liver failure which predisposes individuals to develop hepatic encephalopathy. For these patients, distinguishing between hepatic encephalopathy and Wernicke encephalopathy is a challenge in real-world clinical practice.

A male patient with 21-year medical history of liver cirrhosis presented diarrhea and ascites. One month before this visit, he was noted to have poor appetite and progressive fatigue. After admission, although several major symptoms, including diarrhea, ascites, hyponatremia, and hypoproteinemia, were greatly improved through appropriate treatments, his laboratory indicators were not changed much. His appetite was not reversed at discharge. On the 5th day after discharge, the patient suddenly became reluctant to speak and did not remember the recent happenings. Simultaneously, unsteady gait and strabismus occurred. On the basis of clinical manifestations and brain magnetic resonance imaging scan results, the patient was diagnosed as Wernicke encephalopathy and these relative symptoms were resolved after intravenous vitamin B1.

To our knowledge, this is the second case report of Wernicke encephalopathy developing in a critically ill cirrhotic patient without hepatocellular carcinoma or operative intervention. Wernicke encephalopathy may be underdiagnosed in these patients and this case raises physicians’ awareness of its possible onset.

## 1 Introduction

Wernicke encephalopathy is an acute neurological disorder resulting from thiamine (vitamin B1) deficiency and occurs frequently in patients with compromised absorption, increased metabolism, or increased carbohydrate intake.^[1]^ Delay in its recognition and treatment may lead to significant morbidity, irreversible neurological damage, or even death.^[2]^ Several case reports describe Wernicke encephalopathy as a rare clinical condition; however, it is suggested, by guidelines from the European Federation of Neurological Societies, that Wernicke encephalopathy is not a rare disorder, but rather a rare diagnosis.^[3]^ Here, we present a critically ill patient with decompensated liver cirrhosis who developed Wernicke encephalopathy.

## 2 Consent

Written informed consent was obtained from the patient for the publication of this case report. A copy of the written consent is available for review by the editor of this journal.

## 3 Case report

A 61-year-old man was admitted to the hospital for diarrhea and newly onset ascites. The patient had a 21-year medical history of liver cirrhosis associated with hepatitis B virus (HBV) infection and did not have a habit of alcohol consumption. His serum HBV DNA was not detected 15 years ago. After that, he did not receive any relative examinations or treatments due to economic reasons. One month before the current admission, he was noted to have poor appetite and progressive fatigue. Admission laboratory evaluation yielded the following: white blood cell count, 1.71 × 10^9^; neutrophil percentage, 83.0%; red blood cell count, 3.60 × 10^12^; platelet count, 35 × 10^9^; hemoglobin, 99 g/L; international normalized ratio, 1.51; blood ammonia, 24.7 μmol/L (reference range, 0–30 μmol/L); serum HBV DNA, <40 IU/L; serum alanine aminotransferase, 29 U/L; serum aspartate aminotransferase, 40 U/L; serum alkaline phosphatase, 122 U/L; serum gamma glutamyl transferase, 42 U/L; serum total bilirubin, 27.2 μmol/L (reference range, 3.4–20.5 μmol/L); serum albumin, 26 g/L (reference range, 35–55 g/L); serum creatinine, 92 μmol/L (reference range, 62–115 μmol/L); serum sodium, 130 mmol/L (reference range, 136–145 mmol/L), serum kalium, 3.6 mmol/L (reference range, 3.5–5.2 mmol/L); and serum alpha fetoprotein 2.66 ng/mL (reference range, 0–10 ng/mL). Results of other virologic workups, including tests for hepatitis A, C, D, and E virus, human immunodeficiency virus, and cytomegalovirus, were unremarkable. Abdominal ultrasonography reported liver cirrhosis, portal hypertension, ascites, and splenomegaly. The patient refused to undergo the endoscopy. The neurological examination did not reveal abnormalities.

After admission, his diarrhea was cured by levofloxacin (500 mg daily) at the 3rd day, and ascites was removed through repeated puncture and diuresis at the 6th day. The symptoms of hyponatremia and hypoproteinemia were improved with intravenous supplements; however, the levels of serum sodium and albumin did not arrive at the normal lower limit. At the 7th day, the patient claimed to discharge because of economic reasons and these treatments were thus discontinued. His appetite was always poor from the admission to the discharge. At discharge, his laboratory data were as follows: white blood cell count, 1.23 × 10^9^; neutrophil percentage, 70.3%; red blood cell count, 3.53 × 10^12^; platelet count, 32 × 10^9^; hemoglobin, 101 g/L; international normalized ratio, 1.45; blood ammonia, 17.5 μmol/L; serum HBV DNA, <40 IU/L; serum alanine aminotransferase, 19 U/L; serum aspartate aminotransferase, 39 U/L; serum alkaline phosphatase, 72 U/L; serum gamma glutamyl transferase, 35 U/L; serum total bilirubin, 26.8 μmol/L; serum albumin, 29 g/L; serum creatinine, 96 μmol/L; serum sodium, 134 mmol/L; and serum kalium, 3.9 mmol/L. No mental alteration was observed.

At the 5th day after discharge, the patient suddenly became reluctant to speak and did not remember the recent happenings. Simultaneously, unsteady gait and strabismus occurred. Therefore, he was immediately sent to the local hospital. A brain magnetic resonance imaging (MRI) scan was rapidly performed and the results showed symmetric increased T2 signal in the tectal plate and periaqueductal gray matter. After an urgent telephone consultation to a neurologist and thorough a literature review, Wernicke encephalopathy was eventually diagnosed based on his clinical manifestations and MRI scan results. Intravenous vitamin B1 was therefore prescribed, with 200 mg daily for 5 days. In the days that followed, his memory loss, unsteady gait, and strabismus gradually improved and at the end of the vitamin B1 treatment, these symptoms were resolved. Finally, the patient died of bleeding due to rapid deterioration of his liver function.

## 4 Discussion

**Figure 1:**
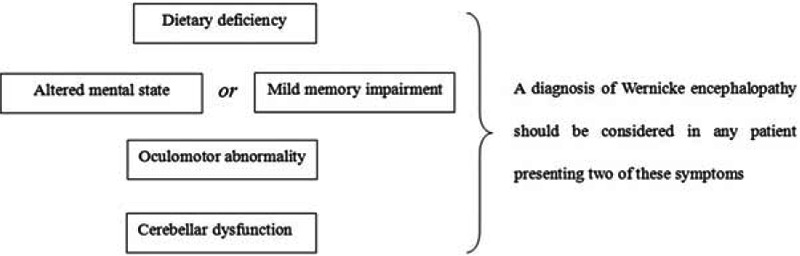
Caine criteria for diagnosis of Wernicke encephalopathy.

Wernicke encephalopathy is a clinical emergency. Classically, its clinical triad consisted of confusion, ataxia, and ophthalmoplegia.^[4]^ Although Wernicke encephalopathy is a disease of known etiology, the above triad is not always present, which leads to difficulties in the differential and confirmed diagnosis. It is reported that the diagnosis is confirmed in 0.4% to 2.8% of autopsies, yet may be overlooked in 68% of patients with alcoholism and 94% of patients without alcoholism.^[1]^ The Caine criteria for diagnosis of Wernicke encephalopathy have been demonstrated to be optimal and are summarized in Fig. 1.^[5]^ Till now, only 4 Wernicke encephalopathy cases associated with liver diseases (Table 1) have been reported in the English literature.^[6–9]^

**Table 1:**
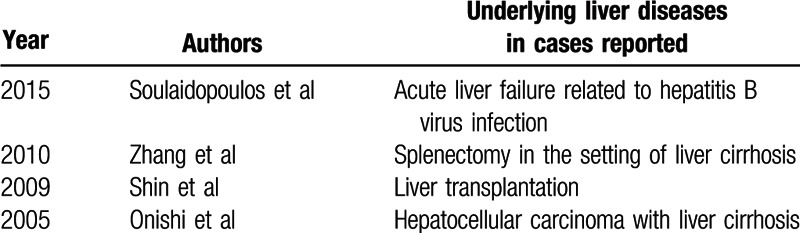
Wernicke encephalopathy associated with liver diseases reported in the English literature.

In this case, insufficient dietary intake (poor appetite) and abnormal glucose metabolism caused by impaired liver function contribute to the development of Wernicke encephalopathy. For patients with underlying liver cirrhosis, distinguishing between hepatic encephalopathy and Wernicke encephalopathy sometimes becomes a tough problem, especially in an emergency.

As is known, hepatic encephalopathy, which may be associated with the increased ammonia or endozepine and is characterized by a wide spectrum of psychiatric and behavioral disturbances and motor disorders, is a common complication of liver failure,^[10,11]^ while mental alteration is usually a most noticeable symptom of Wernicke encephalopathy. Early recognition and diagnosis of Wernicke encephalopathy is pivotal for the prognosis of this medical emergency. If overlooked, it can develop into Wernicke–Korsakoff syndrome, coma, or even death.^[12–15]^ It is vitally important that on observing a patient's early symptoms the physician immediately suspects that the symptoms may point to Wernicke encephalopathy. Factually, distinguishing between hepatic encephalopathy and Wernicke encephalopathy in real-world clinical practice is very difficult. First, an absolute majority of hepatologists are not familiar with Wernicke encephalopathy. Second, no mental alteration is unique in the 2 disorders. Third, distinctions in the neuroimaging findings between the 2 disorders are not easily identified. Like MRI findings in Wernicke encephalopathy, MRI scans in several cases with hepatic encephalopathy also present with symmetrical T2 high signal intensities in the bilateral cerebellar hemispheres and brachium pontis.^[16]^ Regarding the present case, some physicians who participated in the discussion insisted on their diagnosis of hepatic encephalopathy and they did not give up their opinions until the condition of the patient was reversed by intravenous vitamin B1. Therefore, when difficulties exist in distinguishing between hepatic encephalopathy and Wernicke encephalopathy, intravenous vitamin B1 (200 mg) can be considered as a discriminative method or a preemptive treatment.

In conclusion, to our knowledge, this is the 2nd case report of Wernicke encephalopathy developing in a critically ill cirrhotic patient without hepatocellular carcinoma or operative intervention. Wernicke encephalopathy may be underdiagnosed in these patients because it is not easily distinguished from hepatic encephalopathy. This case can also raise physicians’ awareness of the risk of incident Wernicke encephalopathy in critically ill patients with liver diseases.

## Acknowledgments

The authors thank National Natural Science Foundation of China (No. 81501652) for the support.
